# Editorial: Antibody Fc Engineering: Towards Better Therapeutics

**DOI:** 10.3389/fimmu.2018.02450

**Published:** 2018-10-24

**Authors:** Cheng Lei, Rui Gong, Tianlei Ying

**Affiliations:** ^1^Key Laboratory of Medical Molecular Virology of Ministries of Education and Health, School of Basic Medical Sciences, Fudan University, Shanghai, China; ^2^CAS Key Laboratory of Special Pathogens and Biosafety, Wuhan Institute of Virology, Chinese Academy of Sciences, Wuhan, China

**Keywords:** antibody Fc, antibody engineering, therapeutic antibody, Fc receptor, effector function

Efficacy of monoclonal antibodies (mAbs) attributes to both the antigen-binding fragment (Fab) and crystallizable fragment (Fc). Fc, which naturally exists in mAbs but also functions as skeleton in Fc-fusion proteins, can be engineered to possess unique properties and various applications. This collection combines six reviews (Li et al.; Saxena and Wu; Liu et al.; Yang et al.; Ha et al.; Jiang et al.) with three original research articles (Wang et al.; Ye et al.; Okagawa et al.) together to provide substantial knowledge of Fc engineering approaches to regulate effector functions, to extend serum half-life by modification of neonatal Fc receptor (FcRn) binding, to heterodimerize of Fc for design of new Fc formats and to monomerize Fc for improved druggability and novel applications.

Two reviews thoroughly offer an extensive perspective on traditional Fc engineering methods, namely glycoengineering and site mutagenesis (Li et al.; Saxena and Wu). These two methods affect mAbs effector functions by modulating the Fc-FcγRs and FcRn interactions. To affect these interactions, glycoengineering and site mutagenesis both alter either Fc conformations or interfaces of interaction. Li et al. give a detailed review of the biology of IgG-Fc N-glycans (their structures, biosynthesis, and efficacy on mAbs effector functions), the strategies to re-model glycosylation (host cell engineering and chemo-enzymatic glycosylation remodeling), and the discussion of two novel mAbs formats (aglycosylated mAbs and Fc glycan specific antibody-drug conjugates). In the other review, Saxena et al. discuss the modulation of mAbs effector functions and serum half-life (Saxena and Wu). To begin with, a detailed description is given to present differences among Fc receptors. Then Saxena et al. summarize the strategy of modulation of mAbs effector function and pharmacokinetics by Fc engineering. At the end of their article, recent Fc engineering-based mAbs under clinical trials are extensively reviewed.

Currently, apart from Fc engineering at certain amino acids, novel Fc variants are designed to meet the requirements of new antibody scaffolds. The novel variants could derive from recent technologies, such as display-based strategies. Two reviews and one original research article highlight the intense interest in the development of monomeric and heterodimeric Fc (Liu et al.; Ha et al.; Wang et al.) There are two side effects that hinder the application of Fc-fusion proteins. The first side effect is the homodimeric nature of IgG1 Fc; the other is non-specific binding of Fc variants.

In order to solve the two problems above, heterodimeric and monomeric Fc are designed. Heterodimeric Fc is designed to address the heavy chain mispairing issue of bispecific antibodies (bsAb) while retaining biophysical and biological properties of the wild-type Fc. Ha et al. firstly focus on the literature of the design and application of heterodimeric Fc (Ha et al). There are four approaches to design the heterodimeric Fc, namely (i) symmetric-to-asymmetric steric complementarity design (e.g., KiH, knobs-in-holes), (ii) charge-to-charge swap (e.g., DD-KK), (iii) charge-to-steric complementarity swap plus additional long-range electrostatic interactions (e.g., EW-RVT), and (iv) isotype strand swap [e.g., strand-exchange engineered domain (SEED)]. Noteworthily, the pioneering KiH approach's patent is expired and, as a result, widely implemented in current clinical trials. Two different antigen binders could attach to heterodimeric Fc at the N- and/or C-terminus of each Fc chain. This contributes to the creation of various heterodimeric Fc-based antibodies with divergences in specificity, binding valency and binding geometry. Ha et al. then describe a promising scaffold for the next generation of Fc-fusion proteins and cytokines. The authors specified that, in tumor-targeting IgG-based immune-cytokines, in comparison with homodimeric Fc-fused cytokines, the heterodimeric Fc-fused cytokines could reduce the associations with immune cells, which substantially lead to tumor tissue accumulation. This process minimizes systemic toxicity, and further facilitates their development as therapeutics.

Wang et al. report monomeric Fc with half size of IgG1 Fc, significantly lower non-specific binding while retaining FcRn binding (Wang et al.). The phage display library-based technology was utilized to combine rational with random scanning mutagenesis of Fc residues which have been previously identified to impact the Fc dimerization or FcRn binding by the same group. In addition to their latest effort to acquire monomeric Fc, Protein G magnetic beads were introduced to exclude the non-specific binders among the Fc variants. Therefore, this approach could simultaneously identify the monomeric Fc variants (mFc) with low non-specific binding. Moreover, Wang et al. describe the decisive roles of T366R and L368H mutations in creating monomeric status. Importantly, the size of mFc is only half compared with the wild-type Fc while retaining FcRn binding. Consequently, the efficacy of mFc-based fusion proteins and antigen binders could benefit from enhanced tissue penetration and wider range of potential targets.

Recently, some small antibody fragments (e.g., nanobody, human single-domain Ab, scFv, Fab, BITE) and bsAb fragments are designed that have the ability to penetrate better into tissues compared to IgG. However, their small size leads to shorter serum half-life. Besides, these novel formats, derived from IgG, suffer from low solubility and aggregation. Thus, Fc engineering could provide a platform to solve the druggability issues of novel antibody fragments. For instance, the engineered monomeric Fc could be a potential solution based on the minimized non-specific binding, high solubility, high yield, high thermostability, and long *in vivo* half-life. Liu et al. also review recent advances in the therapeutic potential of bispecific molecules and small novel Ab fragments (Liu et al.). The authors then summarize two key approaches to optimize bsAb, namely structural and physicochemical optimization. In another article reviewed by Yang et al. the authors provide the overview on strategies applied on engineering physicochemical properties of Fc.

Regarding to Fc engineering, only a considerably small portion of Fc-fusion proteins have been approved by FDA to date. Fewer Fc-based antigen binders are in clinical studies. However, according to our collection, Fc engineering have become critical tools in both traditional and novel scaffolds of mAbs, and more regulatory approval of Fc engineering-based therapeutics would be expected shortly.

## Author contributions

TY, RG, and CL wrote the manuscript and approved it for publication.

### Conflict of interest statement

The authors declare that the research was conducted in the absence of any commercial or financial relationships that could be construed as a potential conflict of interest.

